# Motor Planning in Chronic Upper-Limb Hemiparesis: Evidence from Movement-Related Potentials

**DOI:** 10.1371/journal.pone.0044558

**Published:** 2012-10-01

**Authors:** Philip John Ainsley Dean, Ellen Seiss, Annette Sterr

**Affiliations:** Department of Psychology, University of Surrey, Guildford, United Kingdom; University of Bologna, Italy

## Abstract

**Background:**

Chronic hemiplegia is a common long-term consequence of stroke, and subsequent motor recovery is often incomplete. Neurophysiological studies have focused on motor execution deficits in relatively high functioning patients. Much less is known about the influence exerted by processes related to motor preparation, particularly in patients with poor motor recovery.

**Methodology/Principal Findings:**

The current study investigates motor preparation using a modified response-priming experiment in a large sample of patients (n = 50) with moderate-to-severe chronic hemiparesis. The behavioural results revealed that hemiparetic patients had an increased response-priming effect compared to controls, but that their response times were markedly slower for both hands. Patients also demonstrated significantly enhanced midline late contingent negative variation (CNV) during paretic hand preparation, despite the absence of overall group differences when compared to controls. Furthermore, increased amplitude of the midline CNV correlated with a greater response-priming effect. We propose that these changes might reflect greater anticipated effort to respond in patients, and consequently that advance cueing of motor responses may be of benefit in these individuals. We further observed significantly reduced CNV amplitudes over the lesioned hemisphere in hemiparetic patients compared to controls during non-paretic hand preparation, preparation of both hands and no hand preparation. Two potential explanations for these CNV reductions are discussed: alterations in anticipatory attention or state changes in motor processing, for example an imbalance in inter-hemispheric inhibition.

**Conclusions/Significance:**

Overall, this study provides evidence that movement preparation could play a crucial role in hemiparetic motor deficits, and that advance motor cueing may be of benefit in future therapeutic interventions. In addition, it demonstrates the importance of monitoring both the non-paretic and paretic hand after stroke and during therapeutic intervention.

## Introduction

Chronic hemiplegia is a common long-term consequence of stroke, affecting 69% of stroke survivors [1]. While good progress has been made towards a better understanding of the mechanisms of recovery and more effective rehabilitation interventions for persons with relatively good residual motor ability [2], [3], [4], much less is known about patients with poor recovery of motor function [5], [6]. This is partly to do with the fact that many studies on motor control in patients focus on motor execution paradigms [7], [8], [9], [10] that rely on the patient’s ability to perform simple movements reasonably well. Moreover, motor recovery is often conceptually equated with motor execution, with less consideration being given to the cognitive processes feeding into the actual motor response. Thus, the majority of studies with stroke patients focus on the endpoint of the motor control process rather than the information processing leading up to the response. While this is a valuable and important approach [11], [12], [13], [14], [15], it neglects the influence of motor cognition on motor behaviour.

Here we argue that in order to obtain a fuller picture of motor control and its recovery after stroke, it is necessary to widen the focus and study paradigms that investigate processes of motor cognition, and to do so across the whole spectrum of motor recovery. The present study therefore aimed to investigate the neural correlates of motor cognition rather than motor execution in a group of patients with sustained poor motor recovery (>1 year post-stroke). Specifically we were interested in characterising the behavioural and neural markers of advanced movement preparation, and examining how these processes are altered for the paretic and the non-paretic arm in people with chronic low-functioning hemiparesis.

Our interest in motor preparation stemmed from two considerations. Firstly, the desire for a robust and well established account of motor cognition, and secondly the utility of a paradigm for studying the motor system that does not rely entirely on motor execution. We therefore used a modified version of the response-priming paradigm [16] which produces robust behavioural effects in healthy controls and, most critically, affords insight into the activation of the cortical motor system through electrophysiological indices obtained before a movement is executed (e.g. [17], [18]). Specifically, this paradigm uses a visual precue that primes participants for a particular movement before the actual movement is required. The precue contains different levels of information on an upcoming response with ‘valid cues’ correctly predicting the subsequently required response (e.g. right hand button press), and ‘ambiguous/neutral cues’ predicting more than one possible response (e.g. left or right hand button press). Behaviourally, this paradigm induces faster response times in valid trials than ambiguous trials, which essentially indicates the benefit of advance movement information on subsequent execution. Electrophysiologically, this effect is associated with the contingent negative variation (CNV), an EEG component that is observed in the interval between the precue and the response cue. The amplitude of the CNV reflects processes of advanced movement preparation and anticipatory attention processes [19], with the late CNV amplitude being modulated by the amount of advanced information provided by the precue [17], [20], [21]. The late CNV is therefore an excellent tool to study the motor system in patients with little residual motor ability. As the CNV reflects both movement preparation and anticipatory attention processes, we modified the standard response-priming paradigm to include a condition where both precue and response cue indicated that ‘no response’ was required. This manipulation was induced as a control condition to account for potential group differences in stimulus processing.

There are relatively few EEG studies on motor preparation with hemiparetic patients, and those that exist have used different methods and reported variable results. Two studies investigated the readiness potential elicited by uncued self-initiated movements and its association with paretic and non-paretic hand movement in the early [22] and chronic [23] stage of recovery. Platz and colleagues [22] reported a diminished readiness potential amplitude with a more lateral and anterior distribution in mild to moderate hemiparesis, whereas Wiese and colleagues [23] found no difference between readiness potentials in patients with chronic mild hemiparesis and matched controls. Crucially, only Verleger and colleagues [24] investigated external stimulus-triggered movement preparation and execution with the response-priming paradigm in a sample of 13 patients with chronic mild hemiparesis. They reported a decrease in CNV amplitude, with similar effects for the paretic and the non-paretic arm. Critically, the authors found no difference in reaction time between patients and controls, which is indicative of excellent motor recovery in these mildly affected patients.

To best of our knowledge no published study has investigated the behavioural and EEG correlates of advanced movement preparation in patients with poor recovery. In an effort to fill this gap the present study used a modified version of the motor priming paradigm [25] in combination with multi-channel EEG to characterise advance movement preparation in a larger sample of stroke patients with moderate-to-severe chronic hemiparesis. In addition to behavioural data, the late CNV was used as electrophysiological marker for functional activity and reorganization of the motor system.

## Methods

### 

#### Patients

Fifty stroke patients presenting with chronic upper-limb hemiparesis (chronicity >1 year [Mean: 4.34±0.51 years; Range: 1–15 years]) following mixed lesions were recruited via local GP’s, hospitals and online support communities. There were 30 left hemiparetic (*HE-L*) patients (Mean age: 53.3±2.3, 15 male, 4 left handed prior to stroke) and 20 right hemiparetic (*HE-R*) patients (Mean age: 54.9±3.0, 11 male, 4 left handed prior to stroke). The terms lesioned and non-lesioned hemisphere, paretic and non-paretic hand will be used throughout this paper in relation to the two patient groups.

Patients had either moderate (48%) or severe (52%) chronic hemiparesis. Level of functioning (Table 1) was determined by the Frenchay Arm Test (FAT; score range: 0 to 5) and the Wolf Motor Function Test (WMFT), which comprises the Functional Ability Scale (FAS; score range: 0 to 7), and Time Taken to do task (TT; maximum 120 seconds, median taken as average). The *HE-L* group had a greater proportion of severe chronic hemiparetic patients (*χ* ^2^ (2, N = 50) = 3.860; *p* = .049) than the *HE-R* group (*HE-L*: proportion = 19/30; *HE-R*: proportion = 7/20).

**Table 1:** Patient demographics.

	Left Hemiparetic (*HE-L*)	Right Hemiparetic (*HE-R*)	Total
**No. Participants**	30	20	50
**Level of function (Severe/Moderate)**	19/11	7/13	26/24
**Age (yrs)**	53.3±2.3	54.9±3.0	53.8±1.8
**Gender (M/F)**	15/15	11/9	26/24
**Chronicity (yrs)**	4.3±0.6	4.2±0.9	4.3±0.5
**WMFT-FAS**	4.0±0.2	4.0±0.3	4.0±0.2
**WMFT-TT (s)**	26.6±6.9	31.6±8.2	28.6±5.2
**FAT**	1.7±0.3	2.1±0.4	1.9±0.3

WMFT: Wolf Motor Function Test; FAS: Functional Ability Score (range: 0 to 7); TT: Time Taken (maximum: 120 s); FAT: Frenchay Arm Test (range: 0 to 5).

Patients were screened for cognitive and emotional problems using a number of questionnaires, and a thorough clinical interview. Patients with clinical levels of depression, seizures within 6 months prior to the experiment, a mini-mental state examination (MMSE) <25, neglect, somatosensory deficits and severe aphasia were excluded.

A healthy control group was recruited (n = 35), from which age and gender matched groups were established for the left (*CO-L*: n = 30, Mean age  = 53.5±2.2, 14 male) and right (*CO-R*: n = 20, Mean age 54.9±2.8, 11 male) hemiparetic groups respectively. All control participants were right handed.

The study was approved by the NHS Ethics Committee, and given a favourable opinion by the Ethics Committee of the University of Surrey. Written informed consent was obtained prior to participation, along with General Practitioner (GP) approval for their participation. All participants had normal or corrected to normal vision. Financial reimbursement was given for travel cost and accommodation when necessary.

#### Experimental task and procedure

A modified response-priming paradigm was employed, in which one of four white precue stimuli (S1), [valid left (<<), valid right (>>) or neutral (< >) or no response (><)], presented within an empty line-drawn white circle on a black background was followed by one of three possible response stimuli (S2), represented by a white semicircle appearing within the line-drawn circle [left button press (left white semicircle), right button press (right white semicircle) or no button press (bottom white semicircle)]. Left, right and no response precues always predicted the response correctly. Neutral precues predicted a response, but not the response hand. Full-preparation trials (40%; valid left [20%], valid right [20%]) and neutral trials (40%) were equally likely, with no response trials half as likely (20%). All trial types were randomised within each block.

**Figure 1:**
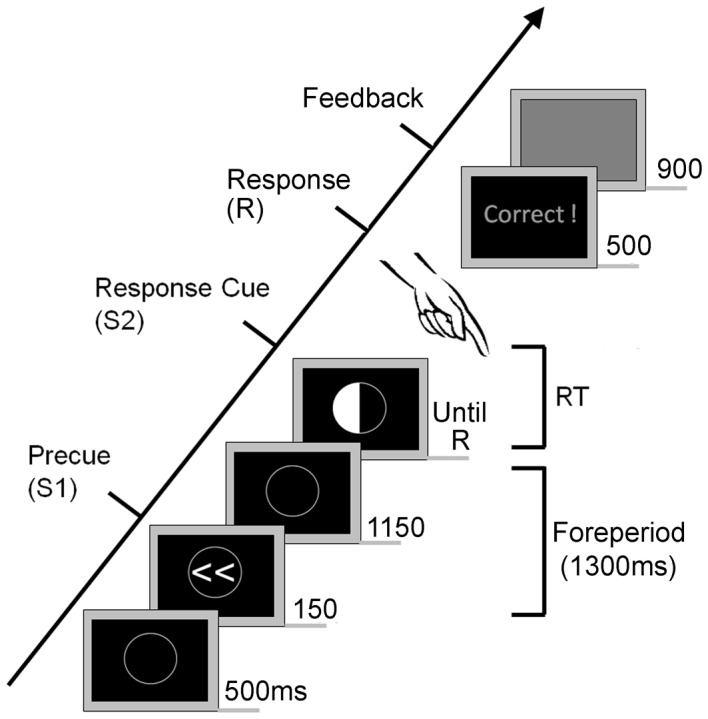
Example trial sequence (valid, left hand response trial). The numbers to the right of the stimuli are the length of time each was presented on screen, in milliseconds. The time given to respond (time between S2 and feedback) varied from 1700 to 3700 ms.

The sequence of events in a trial is illustrated in Figure 1. Trials started with an empty line-drawn circle (fixation circle). After 500 ms, S1 was presented within the circle for 150 ms, followed by an inter-stimuli interval of 1150 ms where an empty line-drawn circle was again presented. The response stimulus (S2) appeared within the circle 1300 ms after S1, and was presented on screen until a response was registered, or until the end of the trial (maximum response time = 1.7 to 3.7 seconds, adjusted for each participant so they could complete a motor response in the majority of trials). Afterwards, feedback was displayed for 500 ms and comprised the following: correct responses (‘Correct!’); incorrect responses (‘Wrong!’; ‘Don’t Respond!’; ‘Too Late!’), and responses within 200 ms of S2 (‘Too Early!’). After feedback presentation the screen turned grey for 900 ms to signal the end of the trial and to allow participants to blink and move their eyes. Participants were instructed to press the response key as fast as possible after viewing the response stimulus.

The participants first undertook some training to familiarise themselves with the procedure. After this, the main experiment consisted of 8 blocks containing 60 trials each. A number of participants (*HE-L*: 11*; HE-R*: 6) did not complete all 8 blocks (Number of Blocks: *HE-L*: Mean 7.2±0.2, Minimum 4; *HE-R*: Mean 7.3±0.3, Minimum 4).

S1 and S2 stimuli were presented within a circle of identical size (2.45°) displayed in white on a black background on a 19″ CRT screen. S1 stimuli within the circle were 0.82° by 1.64° and S2 stimuli were 1.23° by 2.45°. Stimulus presentation and experimental control was implemented with the Neurobehavioural Systems Presentation Software (http://www.neurobs.com/). Responses were executed with the left or right index finger, hand or arm, dependent on ability, using large individual buttons adapted from Test of Attentional Performance (TAP; http://www.psytest.net/OldSite/TAP1.7_uk.html). Large individual buttons were necessary in order to be able to place them where most comfortable for patients to respond to the best of their ability.

#### Electrophysiological recording and processing

The Electroencephalographic (EEG) data was recorded using a 64-channel QuickAmp amplifier (Brain Products; http://www.brainproducts.com/) and Ag/AgCl electrodes positioned according to the international 10-10 system at the sites Fp1, Fp2, Fpz, AF3 to AF4, AFz, F1 to F8, Fz, FC1 to FC6, FCz, FT7 to FT8, FT10, C1 to C6, Cz, T7 to T8, CP1 to CP6, CPz, TP7 to TP10, P1 to P8, Pz, PO3, PO4, PO7, PO8, POz, O1, O2 and Oz. In addition, vertical (VEOG) and horizontal (HEOG) electrooculographic signals were recorded bipolarly using electrodes above and below the left eye and from the outer canthi. Data was recorded in DC mode at 500 Hz against average reference. Electrode impedances were kept below 5 kΩ.

Offline data analysis was performed using BrainVision Analyser Software (Brain Products; http://www.brainproducts.com). The data was low-pass filtered at 25 Hz using a phase-shift free Butterworth filter. Eye movement artefacts were removed through Independent Component Analysis (ICA; [26]). The data were then segmented into condition-specific epochs (trials) of 1400 ms pre to 800 ms post S2. Segments containing other artefacts were rejected using a 20 µV maximum step/sample, ±125 µV absolute segment difference and 0.5 µV minimum activity criterion on all electrodes. For each condition, segments were averaged, 10 Hz low-pass filtered and baseline corrected (baseline: 1400 ms to 1200 ms pre S2) to yield stimulus-locked ERPs. Grand averages were calculated for the identification of components/time-windows chosen for further statistical analysis and for visual display. The late CNV was measured as mean amplitudes for each of the preparation conditions in a 100 ms time window immediately before S2. This window was chosen as it samples the peak of motor preparation activity.

### Data Analysis

#### Behavioural data

Reaction times (RT) and error rates were analysed with mixed-model ANOVAs comprising within-subjects factors CONDITION (Valid, Neutral) and HAND (Paretic, Non-Paretic), and the between-subjects factor GROUP. The first analysis assessed differences between patients and their respective control groups with *Hemiparetic* and *Control* as GROUP factor levels. The HAND factor in controls was matched so that right and left hands were compared to their equivalent in stroke groups. The second analysis assessed differences between right and left paretic patients with *HE-L* and *HE-R* as the GROUP factor levels. For the present paper error rates refer to incorrect responses (“Wrong!”). Errors reflecting early responses (“Too Early!”: reaction time <200 ms) and late responses (responses not given in allotted time, “Too Late!”) are presented in the supporting information (figure S1). Post-hoc independent t-tests were performed as required, with *p* values adjusted for multiple comparisons and (along with degrees of freedom) when the assumption of equality of variances was not met.

#### EEG data

**Figure 2:**
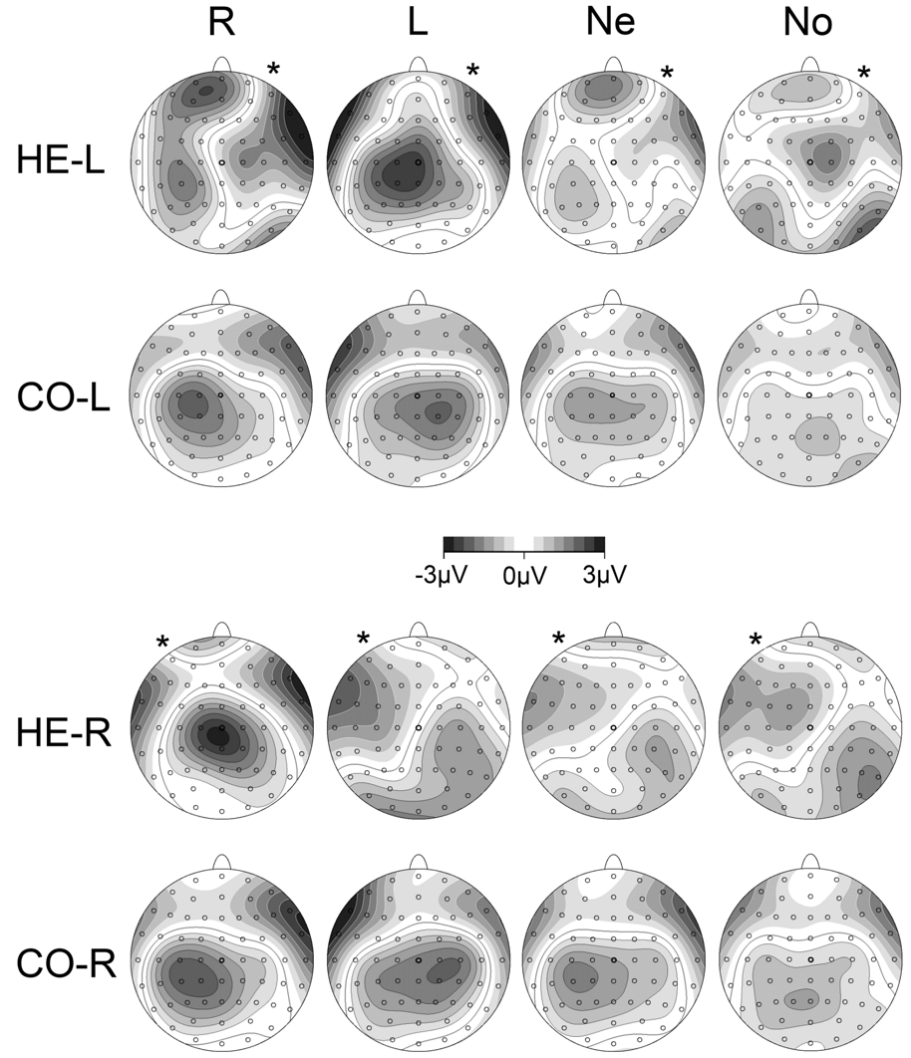
CNV scalp topographies. Time window for map is −100 to 0 ms relative to S2. White contour lines indicate positive activity, black contour lines negative activity. The greyscale fill between the contour lines indicates amount of activation (positive or negative). The small circles on the topographies indicate the electrodes sites, with the thicker circle indicating Cz. The columns indicate: R: Prepare right; L: Prepare Left; Both: Neutral (prepare both hands); No: No Response (no preparation). The rows indicate the four groups studied. Asterisks indicate side of lesion in stroke groups.

For late CNV amplitude analysis, nine electrode clusters of interest were determined through visual inspection of the topographies illustrated in Figure 2. These clusters were in the left hemisphere (FC_Left [FC1+FC3], C_Left [C1+C3] and CP_Left [CP1+CP3]), right hemisphere (FC_Right [FC2+FC4], C_Right [C2+C4] and CP_Right [CP2+CP4]) and midline (FCz, Cz, CPz). Left and right hemispheres were relabelled as ‘lesioned’ and ‘non-lesioned’ dependent on whether *HE-L*/*CO-L* or *HE-R*/*CO-R* were being investigated.

Mixed-model ANOVAs included the within-subjects factors PREPARATION (Valid paretic hand, Valid non-paretic hand, Neutral cue and No Response cue), A-P AXIS (Fronto-Central [FC], Central [C] and Centro-Parietal [CP]) and LATERALITY (midline, lesioned, and non-lesioned). The between-subjects factor of GROUP was either used to compare each stroke group to its matched control (Hemiparetic, Control), or compare the two stroke groups (*HE-L*, *HE-R*). Where the assumption of sphericity was not met, the p value and degrees of freedom were adjusted using the Greenhouse-Geisser method. Bonferroni adjusted pairwise comparisons are also reported.

Separate ANOVAs were calculated for each level of LATERALITY, using the same mixed-model ANOVA format. Subsequent to this, post-hoc independent t-tests were performed for each level of LATERALITY to investigate PREPARATION differences between patients and their matched controls. This analysis was performed on activity averaged across FC, C and CP electrodes within midline, lesioned and non-lesioned hemispheres.

#### Correlation between behaviour and electrophysiology

Pearson correlations were calculated to look at the association of motor preparatory activity with behavioural data in hemiparetic patients (*HE-L*, *HE-R*, n = 50). CNV amplitudes over midline, lesioned and non-lesioned hemispheres measured during preparation of the paretic hand and non-paretic hand were used in the analysis. Behavioural data consisted of reaction times, response priming effects, and error rates for the validly cued paretic and non-paretic hand.

## Results

### Behavioural Data

#### Reaction time

**Figure 3:**
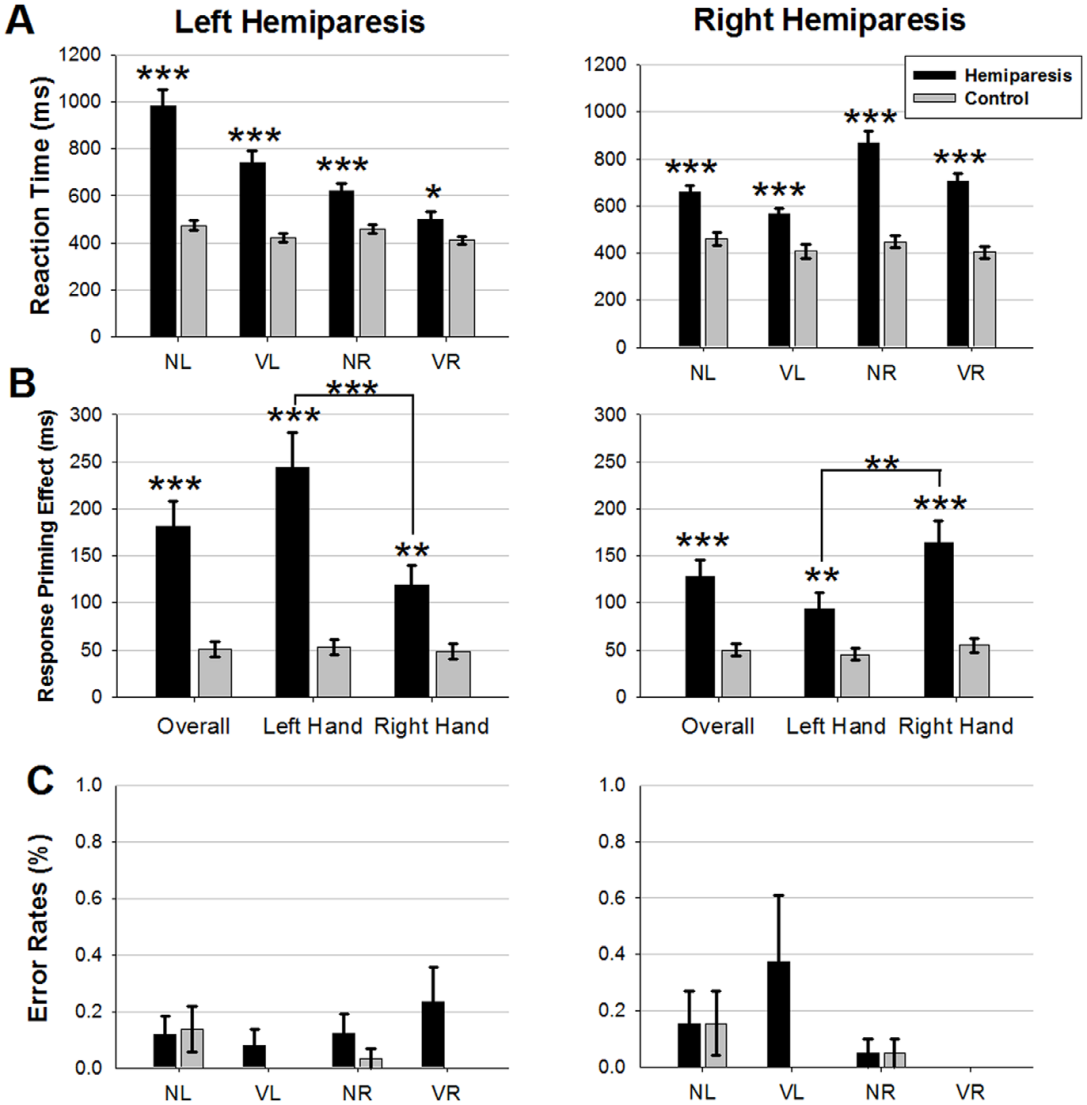
Behavioural data for hemiparetic patients (black) in comparison to controls (grey). A. Reaction times **B.** Response Priming effects **C.** Error rates. VR: validly cued right hand; VL: validly cued left hand; NR: neutrally cued right hand; NL: neutrally cued left hand. Asterisks indicate significant differences using post-hoc independent t-tests (***p<0.001; **p<0.01; *p<0.05).

Reaction time effects are presented in figure 3A. Compared to their respective control groups, reaction times of patients were significantly slower (main effect GROUP: *HE-L:* (*F*(1,58) = 35.8, p<0.001); *HE-R:* (*F*(1,38) = 43.9, *p*<0.001)), and had a larger difference between hands (HAND by GROUP interaction: *HE-L*: *F*(1,58) = 44.1, *p*<0.001; *HE-R*: *F*(1,38) = 37.6, *p*<0.001). Post-hoc t-tests revealed significantly longer reaction times for both hands in *HE-L* participants (paretic hand: *t*(36) = −6.8, *p*<0.001; non-paretic hand: *t*(45) = −3.5, *p*<0.005), and *HE-R* participants (paretic hand: *t*(34) = −7.1, *p*<0.001; non-paretic hand: *t*(38) = −5.2, *p*<0.001).

Response priming effects are presented in figure 3B. Patients showed a greater priming effect (CONDITION by GROUP interaction: *HE-L*: *F*(1,58) = 22.0, *p*<0.001; *HE-R*: *F*(1,38) = 18.3, *p*<0.001) compared to controls. There was also an interaction between CONDITION, HAND and GROUP (*HE-L*: *F*(1,58) = 16.5, *p*<0.001; *HE-R*: *F*(1,38) = 13.6, *p*<0.005), due to the CONDITION by HAND interaction seen in the independent analysis of the patient groups described below.

Independent analysis of hemiparetic groups confirmed the presence of a priming effect (CONDITION: *F*(1,48) = 75.5, *p*<0.001), and slower responses for the paretic hand (HAND: *F*(1,48) = 66.7, *p*<0.001), as well as an interaction between priming effect and hand used (CONDITION by HAND: *F*(1,48) = 24.4, *p*<0.001). Post-hoc analysis revealed a significantly greater priming effect for the paretic hand than the non-paretic hand (*t*(49) = 5.3, p<0.001). In addition, there was a GROUP by HAND interaction (*F*(1,48) = 4.9, *p*<0.05), possibly caused by *HE-L* participants being numerically quicker to respond with their non-paretic hand, and slower to respond with their paretic hand compared to *HE-R* participants. However, there were no overall reaction time differences between *HE-L* and *HE-R* participants (*F*(1,48) = 0.0, *p* = 0.9).

#### Error rates

Error rates are shown in figure 3C. There was no difference between *HE-L* and *HE-R* participants and their matched controls (main effect GROUP: *HE-L: F*(1,58) = 3.1, p = 0.08; *HE-R: F*(1,38) = 1.2, *p* = 0.27), and no difference between the two hemiparetic groups (*F*(1,48) = 0.01, p = 0.94). No other effects or interactions were significant.

### EEG Data

#### Analysis across patient and control groups

The late CNV (−100 ms to 0 ms relative to S2) topographies for the patients and their matched controls are shown in Figure 2. These topographies informed the subsequent analysis of CNV in fronto-central (FC), central (C) and centro-parietal (CP) areas.

Analysis of the hemiparetic groups (*HE-L*, *HE-R*) and their matched controls (*CO-L*, *CO-R*) revealed that CNV amplitude was modulated by PREPARATION condition (*HE-L/CO-L*: *F*(3,174) = 29.3, *p*<0.001; *HE-R/CO-R*: *F*(2,90) = 16.9, *p*<0.001) and these modulations varied between hemispheres (PREPARATION by LATERALITY interaction: *HE-L/CO-L*: *F*(4,250) = 14.2, *p*<0.001; *HE-R/CO-R*: *F*(4,145) = 15.3, *p*<0.001). Furthermore, a main effect of A-P AXIS (*HE-L/CO-L*: *F*(2,96) = 8.0, *p*<0.005; *HE-R/CO-R*: *F*(2, 60) = 10.8, *p*<0.001) was observed, as would be expected with a central/centro-parietal CNV. A difference in CNV amplitude across hemispheres (main effect of LATERALITY) was observed in *HE-L/CO-L* participants (*F*(2,116) = 8.8, *p*<0.001), and approached significance in *HE-R/CO-R* participants (*F*(2,76) = 2.6, *p* = 0.08). Post-hoc comparisons revealed this to be due to a less negative CNV in *HE-L/CO-L* participants across conditions in the lesioned hemisphere compared to midline (mean difference (m.d.) = 0.5, p<0.05) and non-lesioned hemisphere (m.d. = 0.8, p<0.005). Although *HE-R/CO-R* participants exhibited a less negative CNV in the lesioned hemisphere, this was not significantly different when compared to midline (m.d = 0.4, p = 0.13) and non-lesioned hemisphere (m.d = 0.4, p = 0.20).

#### Comparison between patient and control groups

Comparisons between the hemiparetic groups and their matched controls revealed a significant main effect of GROUP for *HE-L* and *CO-L* participants (*F*(1,58) = 4.4, *p*<0.05), and this comparison approached significance in *HE-R* and *CO-R* participants (*F*(1,38) = 3.7, *p* = 0.06). Furthermore, there was a significant GROUP difference in the way CNV amplitude was modulated by preparation condition (GROUP by PREPARATION interaction: *HE-L*: *F*(3,174) = 9.0, *p*<0.001; *HE-R*: *F*(2,90) = 9.9, *p*<0.001) and a GROUP difference in CNV amplitude across hemispheres (GROUP by LATERALITY interaction: *HE-L*: *F*(2,116) = 3.3, *p*<0.05; *HE-R*: *F*(2,76) = 6.1, *p*<0.005). A significant three-way interaction of GROUP, PREPARATION and LATERALITY was observed for *HE-R* and *CO-R* (*F*(4,145) = 2.7, *p*<0.05), and approached significance for *HE-L* and *CO-L* (*HE-L*: *F*(4,250) = 2.3, *p* = 0.051).

These results imply that CNV amplitude differences between the hemiparetic and control GROUPs are modified by a combination of the factors PREPARATION and LATERALITY. In order to understand these results, it is necessary to dissociate the effects of PREPARATION from those of LATERALITY by carrying out post-hoc analysis for each level of one of these factors. Taking into consideration the main effect of LATERALITY seen across hemiparetic groups, and that this seems to be mediated by differences in the lesioned hemisphere, it was deemed appropriate to carry out separate post-hoc ANOVA’s for each level of LATERALITY (*midline*, *lesioned* and *non-lesioned* hemisphere).

#### Comparison between patient and control groups: analysis per hemisphere

The main effects observed in the analysis across groups were again replicated for *midline* (PREPARATION: *HE-L*: *F*(3,174) = 26.2, *p*<0.001, *HE-R*: *F*(2,86) = 13.7; A-P AXIS: *HE-L*: *F*(2,116) = 3.5, *p*<0.05, *HE-R*: *F*(2,76) = 9.0, *p*<0.001) *lesioned* (PREPARATION: [*HE-L*: *F*(2,137) = 27.5, *p*<0.001, *HE-R*: *F*(2,90) = 31.8, *p*<0.001; A-P AXIS: *HE-L*: *F*(2,103) = 5.2, *p*<0.01, *HE-R*: *F*(2,76) = 5.4, *p*<0.01) and *non-lesioned* (PREPARATION: *HE-L*: *F*(3,151) = 17.8, *p*<0.001 *HE-R*: *F*(2,86) = 5.6, *p*<0.005; A-P AXIS: *HE-L*: *F*(2,102) = 5.9, *p*<0.01; *HE-R*: *F*(2,76) = 5.0, *p*<0.01) hemispheres.

**Figure 4:**
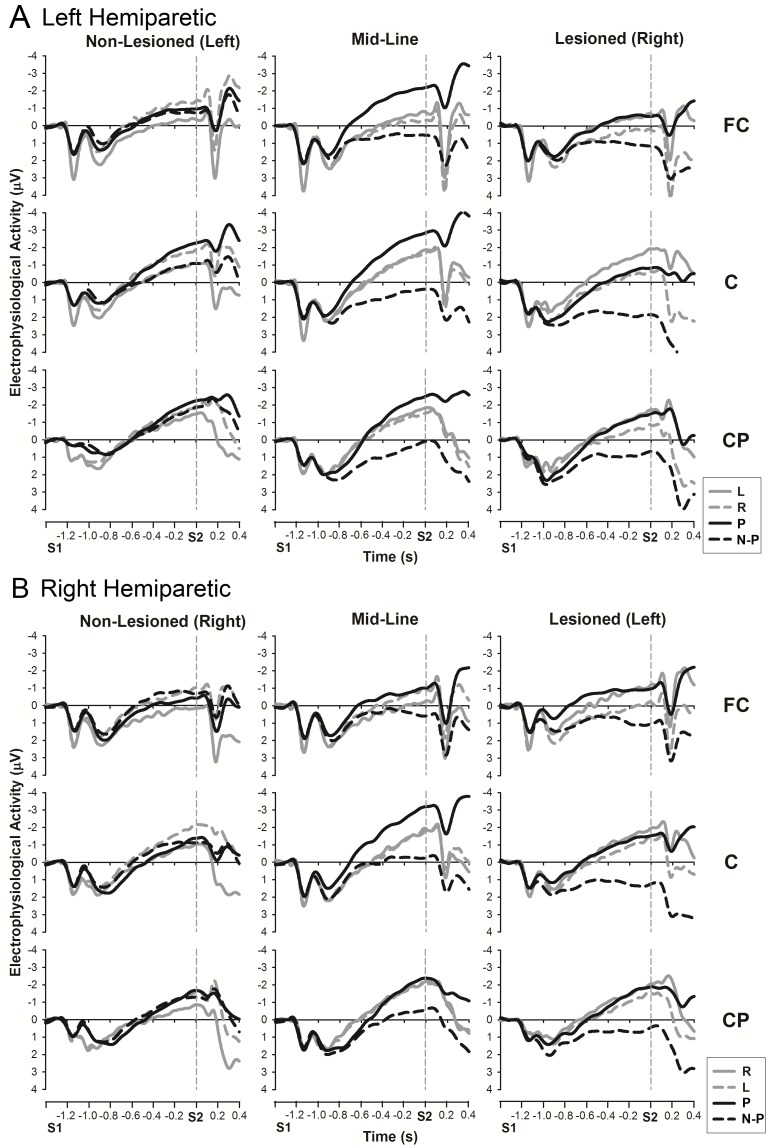
CNV amplitudes for validly cued conditions: comparison between hemiparetic patients (black) and controls (grey). The analysis time window for the late CNV was −100 to 0 ms before S2. **A.** Left hemiparetic (HE-L) patients and matched controls (CO-L). **B.** Right hemiparetic (HE-R) patients and matched controls (CO-R). The word in brackets next to lesioned/non-lesioned indicates the side of lesion in these patients. Legend: P: validly cued paretic hand (solid black line); N-P: validly cued non-paretic hand (black dotted line); R: Validly cued right hand (solid grey line); L: Validly cued left hand (grey dotted line).

**Figure 5:**
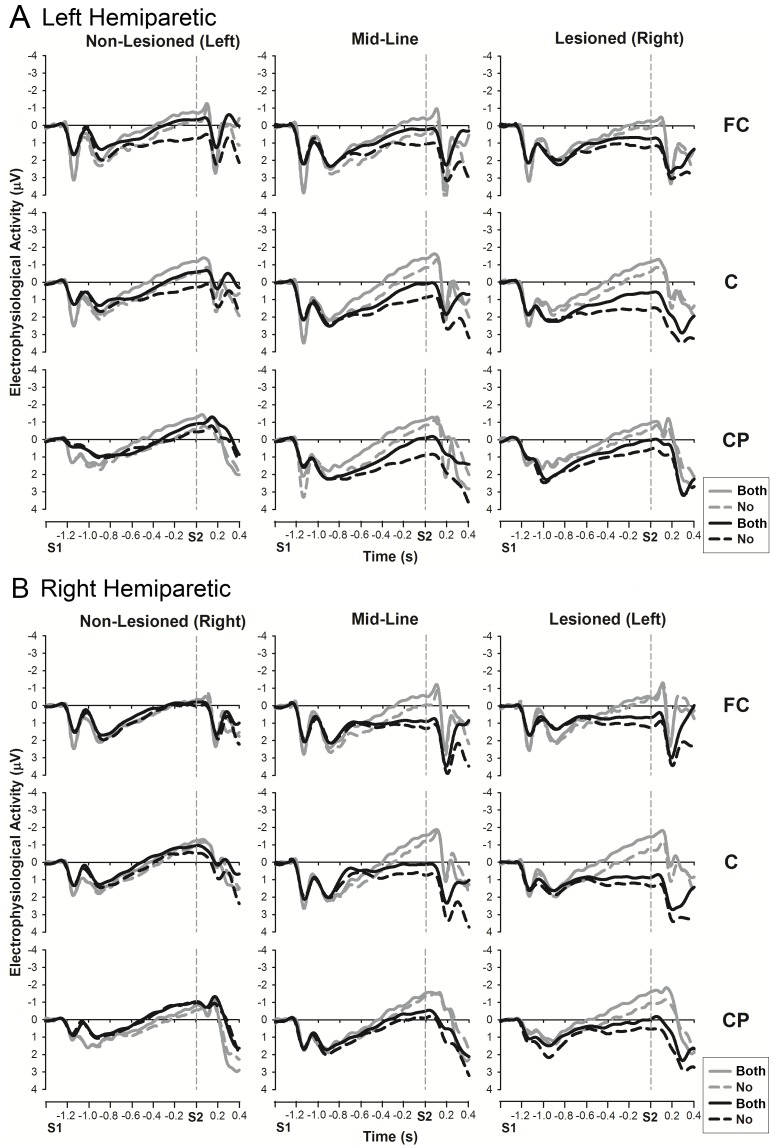
CNV amplitudes for neutrally cued and no response conditions: comparison between hemiparetic patients (black) and controls (grey). The analysis time window for the late CNV was −100 to 0 ms before S2. A. Left hemiparetic (HE-L) patients and matched controls (CO-L). B. Right hemiparetic (HE-R) patients and matched controls (CO-R). The word in brackets next to lesioned/non-lesioned indicates the side of lesion in these patients. Legend: Both: Neutrally cued (both hands); No: No response cued.

A significant difference in CNV amplitude between the hemiparetic groups and their matched controls (main effect of GROUP) was observed over the *lesioned* hemisphere (*HE-L*: *F*(1,58) = 15.3, *p*<0.001; *HE-R*: *F*(1,38) = 11.1, *p*<0.005), whereas there was similar electrophysiological activity over the *midline* (*HE-L*: *F*(1,58) = 2.7, *p* = 0.1; *HE-R*: *F*(1,38) = 2.4, *p* = 0.1) and the *non-lesioned* (*HE-L*: *F*(1,58) = 0.1, *p* = 0.7; *HE-R*: *F*(1,38) = 0.3, *p* = 0.9) hemisphere (see figures 4 and 5). This seems to be the cause of the GROUP by LATERALITY interaction seen in the overall analysis, and indicated by the post-hoc comparisons.

The GROUP difference over the *lesioned* hemisphere was produced by significantly less negative CNV amplitudes in stroke participants when preparing the non-paretic hand (*HE-L*: *t*(58) = 4.4, *p*<0.001; *HE-R*: *t*(38) = 4.5, *p*<0.001) and both hands (*HE-L*: *t*(58) = 3.6, *p*<0.005; *HE-R*: *t*(38) = 3.8, *p*<0.005), or when there was no hand preparation (*HE-L*: *t*(58) = 4.3, *p*<0.001; *HE-R*: *t*(38) = 3.5, *p*<0.005). However, the CNV amplitude when preparing the paretic hand was similar to controls (*HE-L*: *t*(58) = 0.8, *p* = 0.4; *HE-R*: *t*(38) = 0.3, *p* = 0.8).

However, an interaction between PREPARATION condition and GROUP was observed over the *midline* (*HE-L*: *F*(3,174) = 9.3, *p*<0.001; *HE-R*: *F*(2,86) = 9.0, *p*<0.001) and *non-lesioned* hemisphere (*HE-L*: *F*(3,151) = 5.4, *p*<0.005), as well as the *lesioned* hemisphere (*HE-L*: *F*(2,137) = 3.6, *p*<0.05; *HE-R*: *F*(2,90) = 8.6, *p*<0.001). The GROUP by PREPARATION interaction approached significance when comparing *HE-R* to *CO-R* in the *non-lesioned* hemisphere (*HE-R*: *F*(2,86) = 2.5, *p* = 0.08).

Further investigation of these effects revealed findings in *midline* similar to those in the *lesioned* hemisphere, with less negative CNV amplitudes in hemiparetic patients compared to matched controls when preparing the non-paretic hand (*HE-L*: *t*(48) = 2.9, *p*<0.01; *HE-R*: *t*(38) = 2.3, *p*<0.05) and both hands (*HE-L*: *t*(58) = 2.1, *p*<0.05; *HE-R*: *t*(38) = 2.1, *p*<0.05), or when there was no hand preparation (*HE-L*: *t*(58) = 2.7, *p*<0.05; *HE-R*: *t*(38) = 2.5, *p*<0.05). CNV amplitude when preparing the paretic hand was again similar to controls (*HE-L*: *t*(41) = −1.6, *p* = 0.1; *HE-R*: *t*(38) = −1.3, *p* = 0.2). None of the comparisons approached significance in the *lesioned* hemisphere, but looking at figure 4, this effect could be due to a numerically increased CNV amplitude during paretic hand preparation and reduced CNV during non-paretic preparation in *HE-L* participants. Therefore, the cause of the GROUP by PREPARATION interaction seen in the overall analysis seems to be a less negative CNV in both the lesioned hemisphere and midline, when preparing the non-paretic hand, and during neutral an no response conditions. There is no difference in CNV amplitude during paretic hand preparation.

**Figure 6:**
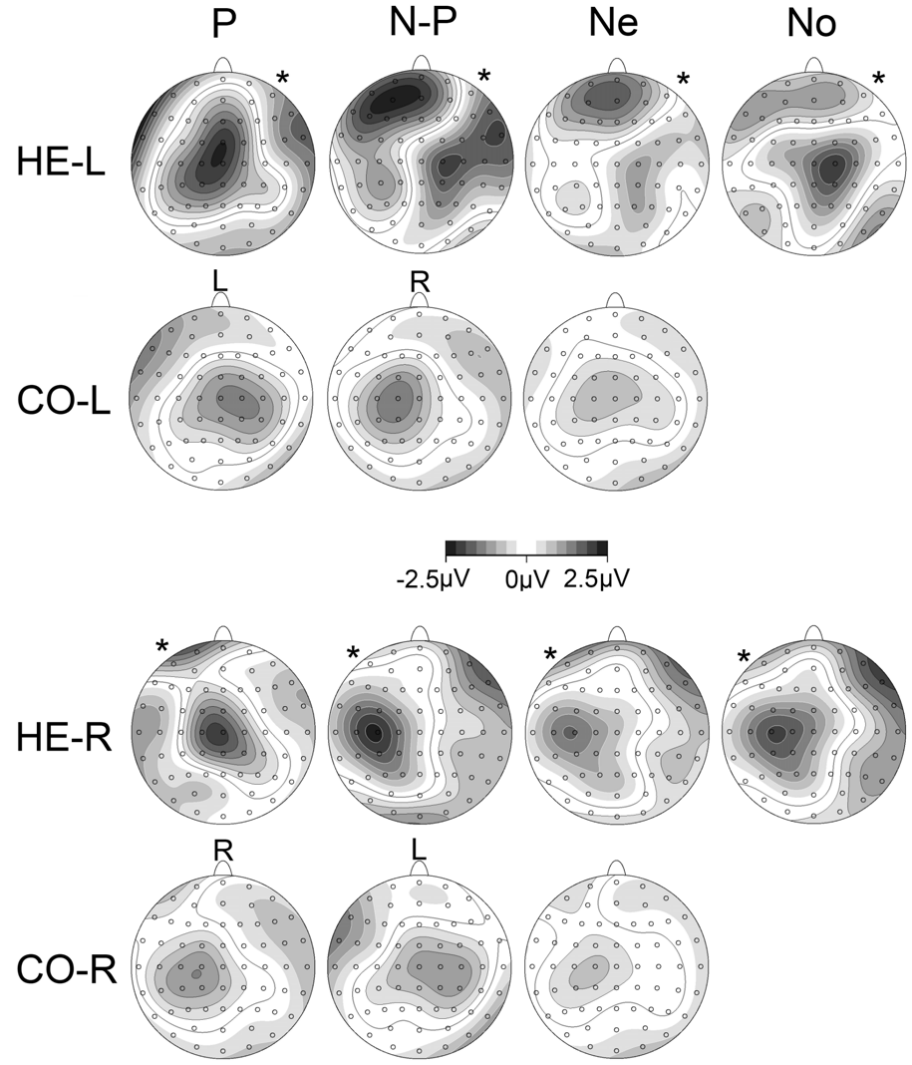
CNV topographies relative to the no response condition. Time window for map is −100 to 0 ms relative to S2. HE-L and CO-L topographies were subtracted from the CO-L no response condition, whereas HE-R and CO-R topographies were subtracted from the CO-R no response condition. White contour lines indicate positive activity, black contour lines negative activity. The greyscale fill between the contour lines indicates amount of activation (positive or negative). The columns indicate: P: Prepare Paretic; N-P: Prepare Non-paretic; Both: Neutral (prepare both); No: No Response (no preparation); R: Prepare Right; L: Prepare Left. The rows indicate the four groups studied. Asterisks indicate side of lesion in stroke groups.

A way of visualising the differences in preparatory activity between hemiparetic groups and controls can be seen in figure 6, which illustrates the electrophysiological potentials for all preparatory conditions (paretic arm, non-paretic arm, neutral and no-response) compared to the no-response condition of the matched control participants. This effectively indicates any increase in motor preparatory or anticipatory attention activity compared to a baseline of stimulus anticipation and attention.

Topographies in control participants reveal contralateral negativity for preparation of left and right hands, and a smaller, more central negativity for the neutral condition (both hands). This pattern is markedly different for the hemiparetic patients who exhibit a very large centralised negativity when preparing the paretic hand, and a large positivity (significantly diminished CNV) over the lesioned hemisphere for the three other conditions. The positivity over the lesioned hemisphere during preparation for the three other conditions is remarkably focused. Another aspect of this illustration is the apparent difference between hemiparetic patients and controls during preparation of the paretic hand, despite there being no overall GROUP difference. There is some indication of a subtle difference in paretic hand preparation that will be further investigated in the individual hemiparetic groups.

#### Comparison between patient groups

**Figure 7:**
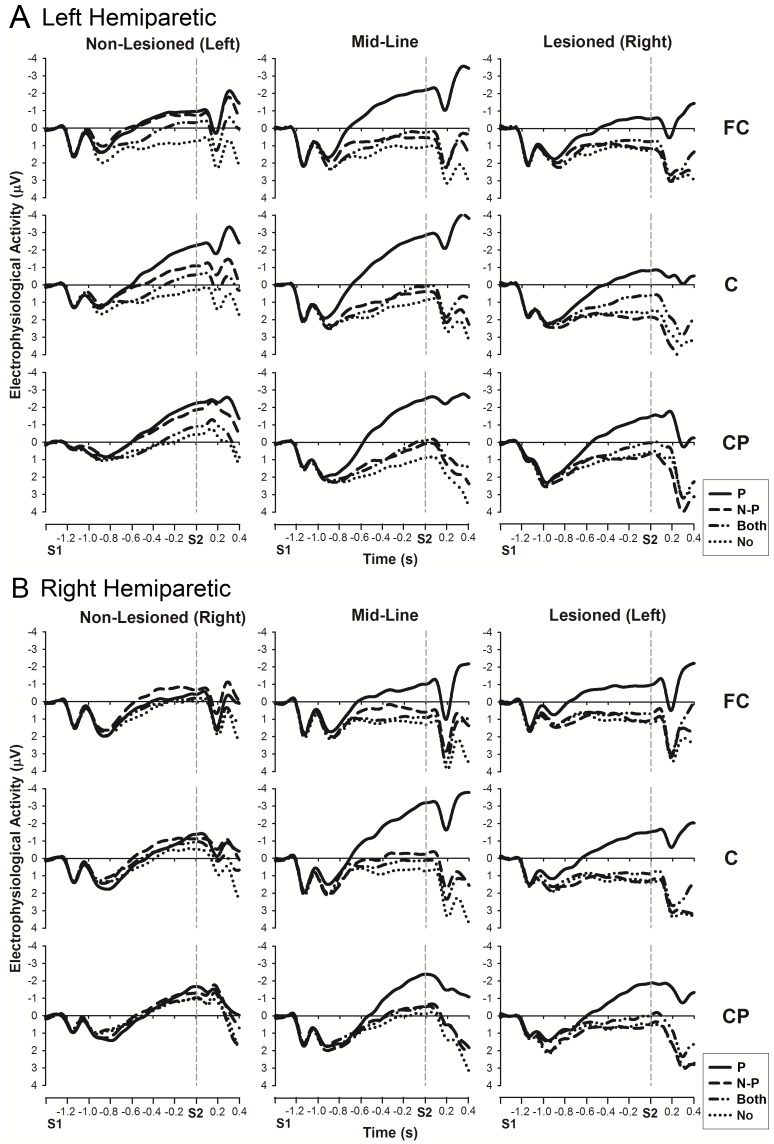
CNV amplitudes for A. left hemiparetic (HE-L) and B. right hemiparetic (HE-R) patients. The analysis time window for the late CNV was −100 to 0 ms before S2. Legend: P: validly cued paretic hand; N-P: validly cued non-paretic hand; Both: neutral (ambiguous cue, prepare both hands); No: no response (no preparation of either hand); L: validly cued left hand; R: validly cued right hand. The word in brackets next to lesioned/non-lesioned indicates the side of lesion in these patients.

Independent analysis of hemiparetic groups (figure 7) replicated the results seen in the analysis across patient and control groups (PREPARATION: *F*(2,118) = 35.3, *p*<0.001, A-P AXIS: *F*(2,77) = 6.6, *p*<0.005, and PREPARATION by LATERALITY interaction: *F*(4,199) = 12.8, *p*<0.001). Furthermore, there was a similar difference between hemispheres (LATERALITY: *F*(2,96) = 11.9, *p*<0.001), caused by less negative CNV in the lesioned hemisphere compared to both the midline (m.d. = 0.7, *p*<0.05) and the non-lesioned hemisphere (m.d. = 1.2, *p*<0.001). There was also an interaction between PREPARATION by A-P AXIS (*F*(4,200) = 3.4, *p*<0.05) not seen in the analysis across patient and control groups.

However, there was no significant difference between the two hemiparetic groups (GROUP: *F*(1,48) = 0.1, *p* = 0.8), even when each LATERALITY was analysed separately (*midline* [*F*(1,48) = 0.1, *p* = 0.819], *lesioned* hemisphere [*F*(1,48) = 0.3, *p* = 0.576], *non-lesioned* hemispheres [*F*(1,48) = 0.0, *p* = 0.985]). No significant interactions with GROUP were found, with only a trend for a GROUP by PREPARATION interaction over the *non-lesioned* hemisphere (*F*(1,48) = 2.6, *p* = 0.07).

Further analysis of the effect of PREPARATION within each LATERALITY revealed that the large midline negativity during paretic hand preparation (seen in figure 6) was substantiated by significantly enhanced CNV over *lesioned* hemisphere and *midline* compared to all other preparation conditions (prepare non-paretic: *lesioned*: m.d. = 2.3, *p*<0.001, *midline*: m.d. = 2.4, *p*<0.001; prepare both hands: *lesioned*: m.d. = 1.7, *p*<0.001, *midline*: m.d. = 2.4, *p*<0.001; no hand preparation: *lesioned*: m.d. = 2.2, *p*<0.001, *midline*: m.d. = 3.1, *p*<0.001). In addition, lower CNV amplitudes were observed over the *lesioned* hemisphere during non-paretic hand preparation in comparison with preparation of both hands (m.d. = 0.6, p<0.05). The PREPARATION by A-P AXIS interaction is likely to be caused by the large midline negativity, across the A-P AXIS during paretic hand preparation, compared with the positive CNV amplitudes seen at fronto-central and central sites during non-paretic hand preparation and preparation of both hands.

In contrast, there was little observable difference between motor preparatory conditions in the *non-lesioned* hemisphere. CNV amplitude during preparation of the non-paretic hand did not differ from that seen during paretic hand preparation (m.d. = 0.3, *p* = 1) or preparation of both hands (m.d. = 0.5, *p* = 0.2), although there was a greater CNV amplitude for paretic hand preparation compared to preparation of both hands (m.d. = 0.8, *p*<0.05). However, CNV amplitudes for all motor preparation conditions were significantly enhanced compared to no hand preparation condition (prepare paretic: m.d. = 1.3, *p*<0.001; prepare non-paretic: m.d.: 1.0, *p*<0.005; neutral: m.d. = 0.5, *p*<0.05), confirming that there was a general level of motor preparation.

Therefore, there does seem to be some subtle changes in preparation activity prior to paretic hand movement in hemiparetic patients, despite no overall GROUP difference compared to matched controls. A larger, more centralised CNV is seen during paretic hand preparation, such that there is no difference in preparation activity between the hands in the non-lesioned hemisphere, and no difference along the A-P AXIS.

### Correlations between Behavioural and Electrophysiological (CNV) Measures in Patients

**Figure 8:**
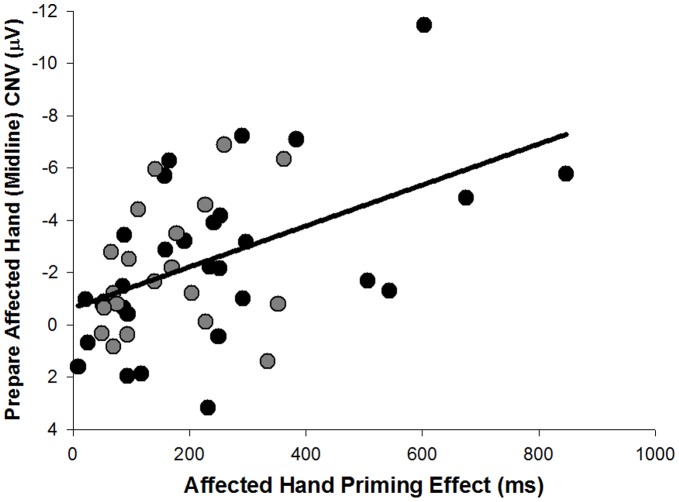
Correlation between CNV during paretic hand preparation and paretic hand priming effect. CNV recorded at midline; paretic hand priming shown for left hemiparetic (black circles) and right hemiparetic (grey circles) patients.

The paretic hand response priming effect was greater in participants with larger midline CNV amplitude during paretic hand preparation (Figure 8; *r*(50) = −0.5, *p*<0.001). When split into left and right hemiparetic groups, the correlation was only seen in *HE-L* (*r*(30) = −0.5, *p* = 0.002), but not *HE-R* participants (*r*(20) = −0.3, *p* = 0.3).

No correlation was observed between midline CNV amplitude during non-paretic hand preparation and paretic hand response priming effect (*r*(50) = 0.1, *p* = 0.6) or non-paretic hand response priming effect (*r*(50) = 0.03, *p* = 0.9).

## Discussion

The present study aimed to characterise the behavioural and neural processes associated with advanced movement preparation of the paretic and non-paretic arm in patients with poor residual recovery. For this purpose we recorded EEG and behavioural data from 50 chronic patients completing a modified version of Rosenbaum’s motor priming paradigm. Behavioural results suggest that (1) the typical response priming pattern, characterised by a facilitation of reaction times for valid compared to neutral cues, is sustained for the paretic and the non-paretic hand in patients despite a general increase in reaction times, and (2) this response priming effect is greater in patients than controls for both hands and larger for the paretic compared to the non-paretic hand. Electrophysiological measures reveal that (4) preparation of the paretic hand is subtly different in hemiparetic patients, with a significantly enhanced midline CNV, despite no overall group difference when compared to controls, (5) preparation of the non-paretic hand and both hands (neutral precue), in addition to anticipatory attention (no response precue), are significantly reduced over the lesioned hemisphere in patients compared to controls, and (6) there is a correlation between larger midline CNV during paretic hand preparation and greater response priming effect (use of precue). There was no significant difference between those patients with right and those with left hemisphere lesions on behavioural and EEG measures.

Taken together, these findings indicate that the principal mechanisms of advanced movement preparation are maintained for the paretic hand even in patients with poor residual motor recovery. Furthermore, it appears that advanced information cues are used to a greater degree to facilitate motor preparation and execution. These results are discussed in detail below.

### Behavioural Data

The response priming effects suggest that the provision of advance movement information may offer a greater benefit to hemiparetic patients than control participants. This finding resonates well with our clinical observation that cuing appears to facilitate practice-based motor rehabilitation. Our data therefore provides initial evidence that is interesting and potentially very relevant for clinical practice. Moreover, patients show similar error levels to controls which negates a speed-accuracy trade off as a simple explanation for the enhanced priming effect observed for the paretic hand. Rather, we suggest that the enhanced priming reflects compensatory mechanisms that might facilitate performance of the paretic hand, but also alter the preparation and performance of the non-paretic hand. This interpretation will be discussed further in the context of the EEG findings.

### Electrophysiological Data

In general, CNV amplitudes measured over the midline and non-lesioned hemisphere were comparable for patients and controls. For the lesioned hemisphere, the CNV amplitudes when preparing the paretic hand were similar in magnitude for patients and controls, but were significantly reduced compared to controls for all other preparatory conditions (prepare non-paretic hand, neutral and no-response). These CNV amplitude effects will be discussed below, separately for each hemisphere.

### CNV in the Midline and Non-lesioned Hemisphere

There was no significant difference between patients and controls in the midline and non-lesioned hemisphere, for any of the preparation conditions. However, there are changes in CNV topography that reflect subtle differences between patients and controls. Firstly, patients did not demonstrate the typical effect pattern of contralaterally enhanced CNVs which has been previously reported for healthy participants [17], [18], [20], and observed in the control group in this study. The CNV amplitude whilst preparing the non-paretic hand was not significantly different from that observed during paretic hand preparation or preparation of both hands. The similarity of preparatory CNV for all motor preparatory conditions might be explained by a functional reorganisation of the motor system, comparable to that found in previous EEG studies on motor execution after stroke [23], [34], [35], [36], [37], [38], [39]. These studies demonstrated the increased usage of ipsilateral and secondary motor areas during movement of the paretic hand, and similar reorganisation may be affecting motor preparatory processes.

Secondly, there is a large midline CNV over Cz during preparation of the paretic hand, whereas control participants show definite contralateral activity over central and centro-parietal electrodes (Figure 2 and 6). This pattern is consistent with a bilateral organisation of the motor control system in hemiparetic patients described in other studies [7], [8], [9], [10], [40], [41]. Increased midline negativity during motor preparation is associated with anticipated effort during bimanual tasks [7], [8], [42], and has been found in a previous study on anarchic hand syndrome [27]. The study on anarchic hand syndrome concluded that this midline negativity reflected an increased effort in initiating a response caused by an inability to attend to or control processing in the contralateral hemisphere [27]. Based on this literature, one might postulate that the midline negativity found in the present study is indicative of enhanced effort to prepare a movement with the paretic arm. This interpretation is strengthened by a positive association of midline CNV amplitude and the magnitude of the response priming effect (Figure 8). As participants expect responses with the paretic hand to be difficult, they may adapt by preparing to a greater degree with this hand which is reflected by greater neural effort and the recruitment of a more bilateral motor network. If paretic hand preparation requires more effort and involves widespread activation, it may be easier for participants to prepare this response in advance than to affect a more general preparation whilst waiting for the response cue (in neutral trials). Thus, there is electrophysiological as well as behavioural evidence that advance cueing may offer a greater benefit to hemiparetic patients than control participants.

### CNV in the Lesioned Hemisphere

CNV amplitudes during non-paretic hand preparation, preparation of both hands, and no hand preparation were significantly reduced in hemiparetic patients compared to matched controls. The CNV reduction during the no response condition is especially interesting, as it suggests that the observed differences may not be related to explicit motor preparation. One possibility is that these results may be related to the imbalance in anticipatory attention processing seen for the paretic hand [27]. However, CNV amplitudes are not just reduced over the lesioned hemisphere for these conditions, but are generally manifest as relatively strong positive potentials.

An alternative explanation is that there has been a state change in motor processing, such as an imbalance in inter-hemispheric inhibition as seen in previous research on hemiparesis [28], [29], [30], [31], [32], [33]. This imbalance could be expressed either as an increase in active inhibition of M1 in the lesioned hemisphere during conditions where movement of the paretic hand is not explicitly required or as increased tonic (background) inhibition of the lesioned hemisphere.

### The Effect of Lesion Side

No significant differences were found in reaction times, error rates or electrophysiological signal between *HE-L* and *HE-R* patients, with only a statistical trend for a GROUP by PREPARATION interaction over the non-lesioned hemisphere suggesting any difference between the groups. As illustrated in figure 7, *HE-L* displays a more standard pattern of activity in the non-lesioned hemisphere, whereas *HE-R* exhibits relatively little difference between preparation conditions. The observed differences are non-significant, and could be due to the lower participant numbers in the *HE-R* group. There is some evidence for a greater role in bilateral motor control for the dominant hemisphere [44], [45], [46], [47], and lesions in this hemisphere may lead to poorer recovery [35], [43]. However, lesions in the non-dominant hemisphere have been associated with greater inter-hemispheric inhibition after stroke [48], meaning that the role of lesion side in recovery is inconclusive. The current study provides evidence that lesion side has little effect on motor preparatory processes.

### Implications

The behavioural and electrophysiological data presented here suggest that advance movement information modulates motor performance in hemiparetic patients. This finding implies that the strategic use of cues might enhance or hinder rehabilitation interventions. Further research is necessary to determine the facilitatory characteristics of advanced movement information and their implementation in the clinical setting.

An additional implication of this study is that the non-paretic hand is not “unaffected”, but exhibits both behavioural and electrophysiological changes in comparison to controls. Consequently, it is prudent to monitor the neurological activity and behaviour of the non-paretic hand after stroke and during therapeutical intervention. Motor impairments of the non-paretic hand [49], are particularly important in the context of interventions that specifically aim to increase paretic arm use through enforced disuse of the non-paretic limb, such as CIMT [50], [51], [52], [53], [54], [55], [56], [57]. Furthermore, hemiparetic patients did not demonstrate the typical effect pattern of enhanced CNV amplitudes over the non-lesioned hemisphere when preparing the contralateral (non-paretic) hand. This suggests that the non-lesioned hemisphere cannot necessarily be thought of as “intact”, as functional alterations related to the hemiparesis may have occurred. Indeed, previous studies have found that the inhibition of non-lesioned motor areas can be used as a therapeutic intervention for stroke patients [58], [59], highlighting the role of functional changes within this hemisphere in recovery of the paretic hand [31], [60].

### Conclusion

The present study demonstrates enhanced motor preparatory processes but slowed reaction times for both hands in moderate-to-severe chronic hemiparetic patients. Electrophysiological analysis revealed that there was a significantly increased midline CNV in hemiparetic patients during preparation of the paretic hand, despite no overall group difference when compared to controls. Furthermore, this midline CNV was correlated with response priming effect. Taken together, this was thought to be indicative of greater anticipated effort to respond, and suggests that advance cueing of motor response might be of great benefit to hemiparetic patients. All other preparatory conditions (non-paretic hand, neutral, no preparation) were similar for patients and controls in the midline and non-lesioned hemisphere, but significantly reduced in patients over the lesioned hemisphere. The cause of this reduction may be related to similar anticipatory attention changes as those seen for the paretic hand, or could be due to a state change in motor processing (for example an imbalance in interhemispheric inhibition). There is some evidence for functional alteration when preparing the non-paretic hand in both the non-lesioned and lesioned hemisphere, and these may be related to the significant reduction in reaction times in the non-paretic “unaffected” hand. Lesion side does not seem to influence results in this study, but future research could focus on lesion location as patients with cortical lesions (particularly of the primary motor cortex) demonstrate less recovery than those with subcortical lesions [61], [62].

## Supporting Information

Figure S1 **Supplementary error rate data for hemiparetic patients (black) in comparison to controls (grey). A.** Too early error rate **B.** Too late error rate. VR: validly cued right hand; VL: validly cued left hand; NR: neutrally cued right hand; NL: neutrally cued left hand. Asterisks indicate significant differences using post-hoc independent t-tests (***p<0.001; **p<0.01; *p<0.05).
(TIF)

Data S1 **Supplementary results for error rate data in the modified response priming paradigm.**
(DOC)
