# Supplementary figures and images for: Motor Planning in Chronic Upper-Limb Hemiparesis: Evidence from Movement-Related Potentials
Source: PLoS One. 2012 Oct 1;7(10):e44558. doi: 10.1371/journal.pone.0044558 (PMC3462178; doi:10.1371/journal.pone.0044558)

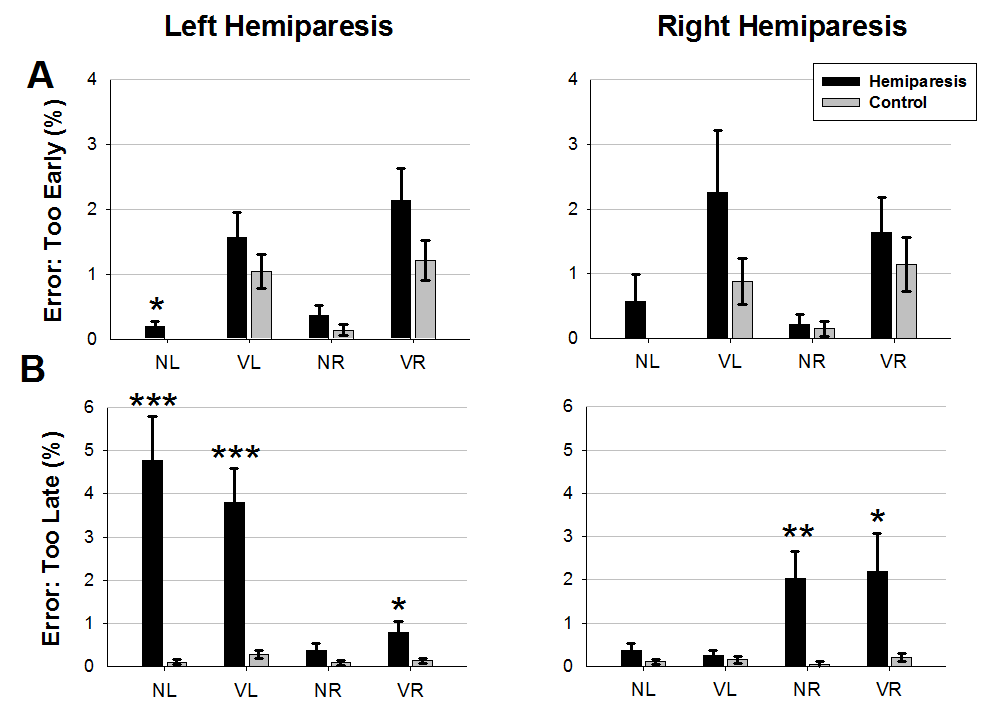

Supplement: Figure S1 — Supplementary error rate data for hemiparetic patients (black) in comparison to controls (grey). A. Too early error rate B. Too late error rate. VR: validly cued right hand; VL: validly cued left hand; NR: neutrally cued right hand; NL: neutrally cued left hand. Asterisks indicate significant differences using post-hoc independent t-tests (***p<0.001; **p<0.01; *p<0.05). (TIF) [file pone.0044558.s001.tif]
