# Supplementary material for: Motor Planning in Chronic Upper-Limb Hemiparesis: Evidence from Movement-Related Potentials
Source: PLoS One. 2012 Oct 1;7(10):e44558. doi: 10.1371/journal.pone.0044558 (PMC3462178; doi:10.1371/journal.pone.0044558)
Supplement: Data S1 — Supplementary results for error rate data in the modified response priming paradigm. (DOC) [file pone.0044558.s002.doc]

**Supplementary Data**

There was no overall difference in early or late response error rates between the two hemiparetic groups (too early: *F*(1,48) = 0.07, p=0.80; late response: *F*(1,48) = 3.4, p=0.07).

*Early Responses* (figure S1A)*.* There was no significant difference in early responses between hemiparetic patients and their matched controls (main effect GROUP: *HE-L: F*(1,58) = 3.6, p=0.06; *HE-R:* *F*(1,38) = 1.9, *p*=0.18), and no significant interactions between GROUP and CONDITION or HAND. However, there was a main effect of CONDITION (*HE-L: F*(1,58) = 34.4, p<0.001; *HE-R:* *F*(1,38) = 13.6, *p*=0.001), with valid responses more likely to elicit early responses independent of group. The main effect of HAND approached significance in the *HE-L/CO-L* comparison (*F*(1,58) = 3.8, *p*=0.06), with more errors seen in the non-paretic/right hand.

*Late Responses* (figure S1B). Hemiparetic patients had significantly more late responses compared to their matched controls (*HE-L: F*(1,58) = 22.4, p<0.001; *HE-R:* *F*(1,38) = 8.2, *p*<0.01). Moreover, the main effect of HAND was significant, along with an interaction between HAND and GROUP (HAND: *HE-L: F*(1,58) = 24.2, p<0.001; *HE-R:* *F*(1,38) = 6.0, *p*<0.05; HAND*GROUP: *HE-L: F*(1,58) = 22.1, p<0.001; *HE-R:* *F*(1,38) = 5.9, *p*<0.05). These results were caused by both hemiparetic groups having significantly more late responses with their paretic hand. The *HE-L*/*CO-L* comparison also revealed a GROUP by HAND by CONDITION effect (*F*(1,58) = 5.1, p<0.05) and the HAND by CONDITION interaction approached significance (*F*(1,58) = 3.4, p=0.07). This is likely to be caused by numerically greater errors when neutrally cued for the paretic hand and when validly cued for the non-paretic hand in hemiparetic patients.
